# Effects of Icariside II on Corpus Cavernosum and Major Pelvic Ganglion Neuropathy in Streptozotocin-Induced Diabetic Rats

**DOI:** 10.3390/ijms151223294

**Published:** 2014-12-15

**Authors:** Guang-Yi Bai, Feng Zhou, Yu Hui, Yong-De Xu, Hong-En Lei, Jin-Xian Pu, Zhong-Cheng Xin

**Affiliations:** 1 Andrology Center, Peking University First Hospital, Peking University, Beijing 100034, China; E-Mails: bgy73kr@163.com (G.-Y.B.); zhoufeng0319@163.com (F.Z.); xyongde@gmail.com (Y.-D.X.); hongenlei@foxmail.com (H.-E.L.); 2 Department of Urology, First Affiliated Hospital of Soochow University, Suzhou 215006, China; E-Mail: huiyfishing@126.com

**Keywords:** erectile dysfunction, diabetes, neuropathy, nNOS, NGF, major pelvic ganglion, Icariside II

## Abstract

Diabetic erectile dysfunction is associated with penile dorsal nerve bundle neuropathy in the corpus cavernosum and the mechanism is not well understood. We investigated the neuropathy changes in the corpus cavernosum of rats with streptozotocin-induced diabetes and the effects of Icariside II (ICA II) on improving neuropathy. Thirty-six 8-week-old Sprague-Dawley rats were randomly distributed into normal control group, diabetic group and ICA-II treated group. Diabetes was induced by a one-time intraperitoneal injection of streptozotocin (60 mg/kg). Three days later, the diabetic rats were randomly divided into 2 groups including a saline treated placebo group and an ICA II-treated group (5 mg/kg/day, by intragastric administration daily). Twelve weeks later, erectile function was measured by cavernous nerve electrostimulation with real time intracorporal pressure assessment. The penis was harvested for the histological examination (immunofluorescence and immunohistochemical staining) and transmission electron microscopy detecting. Diabetic animals exhibited a decreased density of dorsal nerve bundle in penis. The neurofilament of the dorsal nerve bundle was fragmented in the diabetic rats. There was a decreased expression of nNOS and NGF in the diabetic group. The ICA II group had higher density of dorsal nerve bundle, higher expression of NGF and nNOS in the penis. The pathological change of major pelvic nerve ganglion (including the microstructure by transmission electron microscope and the neurite outgrowth length of major pelvic nerve ganglion tissue cultured *in vitro*) was greatly attenuated in the ICA II-treated group (*p* < 0.01). ICA II treatment attenuates the diabetes-related impairment of corpus cavernosum and major pelvic ganglion neuropathy in rats with Streptozotocin-Induced Diabetes.

## 1. Introduction

Erectile dysfunction is a common clinical entity that affects mainly men older than 40 years according to the classical causes of erectile dysfunction, such as diabetes mellitus, hypertension, and several common lifestyle factors [1]. There are various causes for organic erectile dysfunction (ED), most of which involve damage to nerves, blood vessels, corporal smooth muscle, and/or endothelial cells [2,3]. The incidence of ED among men suffering diabetes mellitus (DM) is three times higher than in non-diabetic men. Indeed, up to 75% of men with DM experience ED and onset of the condition typically occurs at an earlier age [4].

The corpora cavernosa are cylindrical vessels that expand with blood during penile erection. If cavernous wall resistance decreases, radius increases and internal pressure decreases according to La Place’s law. It is by this process that venous leak ED occurs. Many factors contribute to the pathogenesis of diabetic ED, including oxygen free radicals, NO-cGMP pathway dysfunction, inflammation, fibrosis, and cavernous neuropathy [5,6,7].

Substantial advances have led to the development of successful oral therapies, namely the phosphodiesterase type 5 inhibitors (PDE5Is), which were currently the first-line treatments of choice for management of ED [1], but men with diabetes-related ED are less responsive to PDE5I [8]. Gene therapy and stem cell therapy are promising treatments that have shown some efficacy in pre-clinical studies [9]. However, neither modality is currently available for wide clinical usage, their ultimate role in management of ED in humans is yet to be determined [10,11,12,13,14].

Plants of the genus Epimedium (Herba epimedii) have been utilized for the treatment of erectile dysfunction in Traditional Chinese Medicine for centuries. Icariin (C_33_H_40_O_15_, molecular weight: 676.67) and icariside II (ICA II, C_27_H_32_O_10_, molecular weight: 514.54), two flavonols isolated from herba epimedii, have a close structural relationship. Icariin is believed to be the principal active moiety of herba epimedii. Xin reported that icariin is a cGMP-specific PDE5 inhibitor, with selective inhibitory activity on PDE5 that is more than 100 times that of PDE4 (PDE4/PDE5 of IC_50_) [15]. Liu reported that Icariin could improve erectile function by preserving smooth muscle, endothelium content and nNOS expression in the penis of STZ-induced diabetic rats; TGFβ1/Smad2 signaling pathway might play an important role in icariin improving erectile function in diabetic rats [16]. Zhou reported that ICA II could alter corpus cavernosum fibrous-muscular pathological structure in DM rats that could be regulated by the TGFβ1/Smad2/CTGF and NO-cGMP signaling pathways [17]. 

Neuronal nitric oxide synthase (nNOS) is an important enzyme involved in the production of nitric oxide (NO) and thus regulates penile vascular homeostasis; molecular mechanisms involved in regulation of these NOS isoforms in the development of ED are essential to discovering the pathogenesis of ED in various disease states [18]. Shindel *et al.* [19] reported that icariin led to significantly greater neurite length of major pelvic ganglia (MPG) fragments *in vitro*. In the same study, the authors reported improved penile hemodynamics and up-regulation of cavernous nNOS expression in icariin-treated rats relative to the neurotrophic effects.

In this study we administered ICA II to the diabetic rats. Rats underwent functional testing of erectile hemodynamics during cavernous nerve stimulation as well as histological and molecular assessment of penile tissues for investigating the effects of ICA II on improving corpus cavernosum pathological change of dorsal nerve bundle and the major pelvic nerve ganglion.

## 2. Results

### 2.1. Metabolic Variables and ICP/MAP (Intracavernous Pressure/Mean Arterial Pressure) Assessment

**Figure 1:**
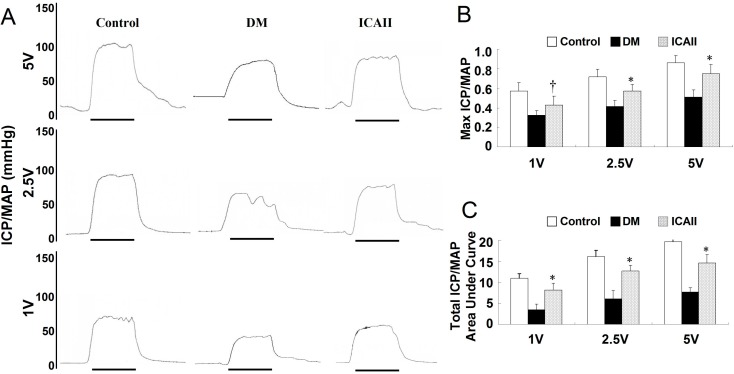
ICA II enhances erectile function in diabetic rats. (**A**) Representative intracavernous pressure (ICP) responses to electrical stimulation of the cavernous nerve for age-matched normal control and diabetic groups. The stimulus interval (60 s) is indicated by a solid bar; (**B**,**C**) Erectile function presented as maximal ICP/MAP and total ICP/MAP (AUC/MAP) in each group. * *p* < 0.01, **^†^** *p* < 0.05 in the ICA II-treated group compared with the DM placebo group.

There were no significant differences in body weight or serum glucose concentrations between the three groups at baseline. Twelve weeks after diabetes was induced, the fasting glucose concentrations of diabetic animals were significantly higher than concentrations in the age-matched normal controls. Body weight was significantly lower in the diabetic rats than in the control group. No significant difference in body weight or glucose concentration was found between the DM and ICA II-treated groups (Table 1). All erectile function variables, including ICP/MAP ratio and ICP area under the curve (AUC)/MAP, were significantly lower in the diabetic rats (Figure 1).

**Table 1:** Metabolic and physiological variables.

Variable	Control	Diabetic	ICA II
Initial weight (g)	219.6 ± 25.1	221.2 ± 13.4	211.4 ± 17.7
Final weight (g)	602.1 ± 23.2	346.1 ± 32.6	397.5 ± 24.4
Initial fasting glucose (mg/dL)	109.2 ± 3.6	107.2 ± 3.2	105.7 ± 2.6
Final fasting glucose (mg/dL)	112.4 ± 11.5	493.6 ± 32.3	423.5 ± 22.5
MAP (mm Hg)	109.27 ± 7.15	96.22 ± 5.85	104.25 ± 4.20
ICP (mm Hg)	96.33 ± 8.30	41.32 ± 7.55	74.30 ± 9.33 ^#^
AUC	1980 ± 135	675 ± 156	1667 ± 142 ^#^

Values are the mean values (±standard deviation) from *N* = 20 animals per group. ^#^ *p* < 0.0001 compared with the DM placebo group. Ratios of maximal ICP (mmHg) and AUC to MAP (mmHg) were calculated to normalize for variations in systemtic blood pressure. ICP = intracavernous pressure; MAP = mean arterial pressure; AUC = area under the curve.

### 2.2. The Pathological Change of the Dorsal Nerve Bundle

**Figure 2:**
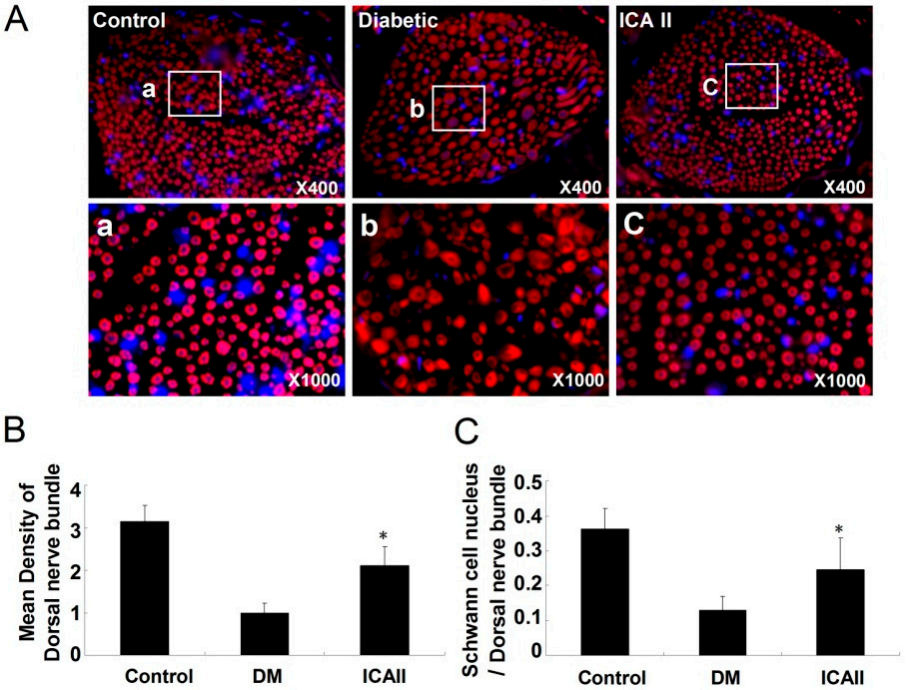
Semiquantitative analyses of dorsal nerve bundle and Schwann cell nucleus in normal, diabetic and ICA II rat corpus cavernosum groups (a/b/c showed the typical area of the three groups respectively). (**A**) Immunofluorescence stained with antibody to Neurofilament (NF); (**B**) The mean density of dorsal nerve bundle by immunofluorescence staining; (**C**) The ratio of Schwann cell nucleus to dorsal nerve bundle. Each bar depicts the mean values (±standard deviation) from *N* = 10 animals per group. * *p* < 0.01 in the ICA II-treated group compared with the DM placebo group.

We performed immunofluorescence staining with antibody to neurofilament to assess the dorsal nerve bundle content (Figure 2). The penis tissue from diabetic rats showed axonal swelling, axonal vacuolization and less density of neurofilament in the cavernous dorsal nerve bundle relative to normal controls. Animals that had been treated with ICA II had significantly greater density of neurofilament staining and better regular structure (less axonal swelling, axonal vacuolization and higher density of neurofilament (Figure 2B). Also we found that the ratio of Schwann cell nucleus/dorsal nerve bundle was significantly lower in the diabetic group compared to the normal control and ICA II-treated group (Figure 2C).

**Figure 3:**
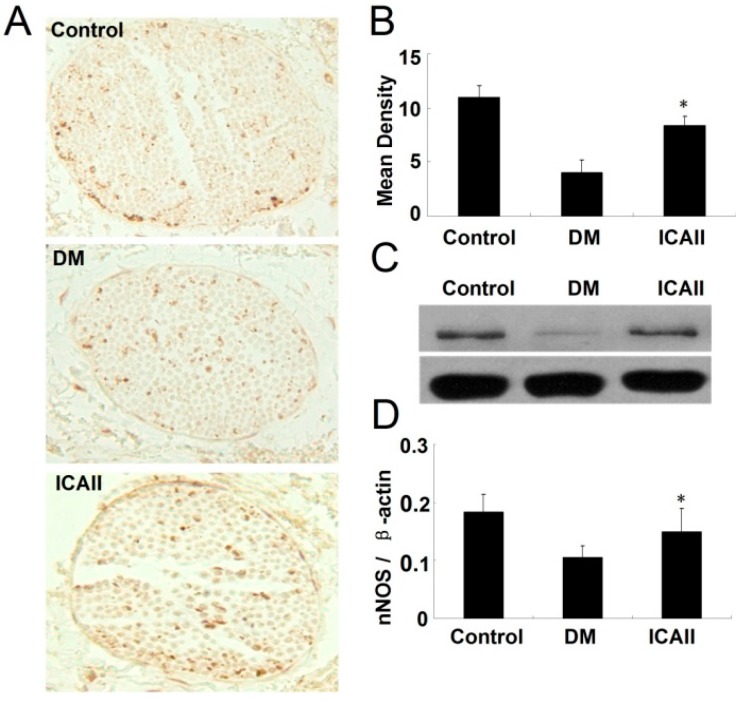
ICA II enhances the nNOS expression in diabetic rat corpus cavernosum. (**A**) Immunohistochemical staining with antibody to nNOS in dorsal nerve bundle of normal and diabetic rat; (**B**) Semiquantitative analysis of nNOS protein expression; (**C**) Western blots analysis of the nNOS expression in normal and diabetic rat corpus cavernosum; (**D**) The ratio of nNOS to β-actin by Western blot. Each bar depicts the mean values (±standard deviation) from *N* = 10 (immunohistochemical staining) and *N* = 4 (Western blots) animals per group. * *p* < 0.01 in the ICA II-treated group compared with the DM placebo group.

We performed nNOS immunohistochemical staining to locate and quantify NOS-positive nerve fibers content in each group. The number of nNOS-positive nerve fibers in the dorsal penile nerves was significantly greater in the ICA II-treated group compared to the untreated diabetic animals (Figure 3A,B).

We performed Western blots to evaluate the expression of nNOS in the penis tissue of each group. The nNOS protein expression was significantly lower in the DM placebo group compared to the normal control group, however, the level of the protein expression was significantly greater in the ICA II-treated groups relative to the untreated diabetic group (Figure 3C,D).

**Figure 4:**
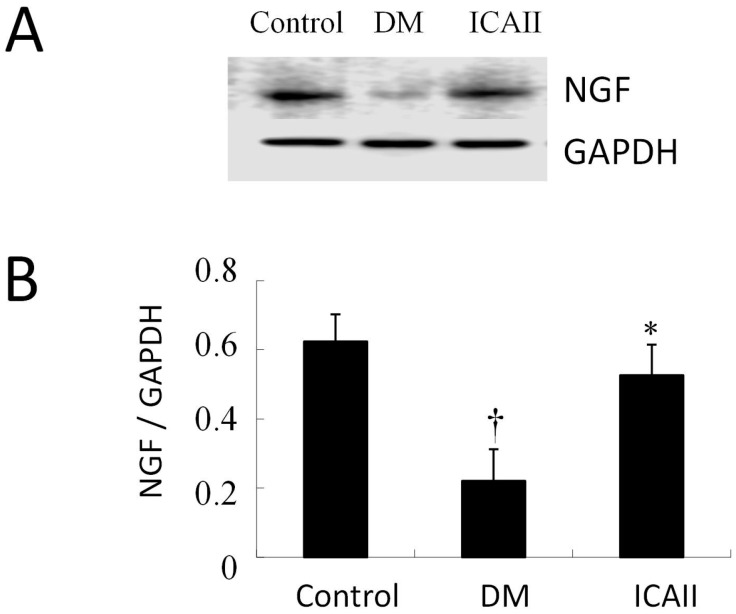
ICA II enhances NGF expression in the corpus cavernosum. (**A**) Western blot analysis of NGF expression in normal and diabetic rat corpus cavernosum; (**B**) Semi-quantitative analysis of NGF protein expression in testes with Western blot. ^**†**,^* *p* < 0.01 in the ICA II-treated group compared with the Control and DM placebo group respectively.

We also performed Western blots to evaluate the expression of NGF (nerve growth factor) in the penis tissue in each group. NGF protein expression was significantly lower in the DM placebo group compared to the normal control group; and it was significantly greater in the ICA II-treated groups relative to the untreated diabetic group (Figure 4).

### 2.3. The Pathological Change of Major Pelvic Ganglion

**Figure 5:**
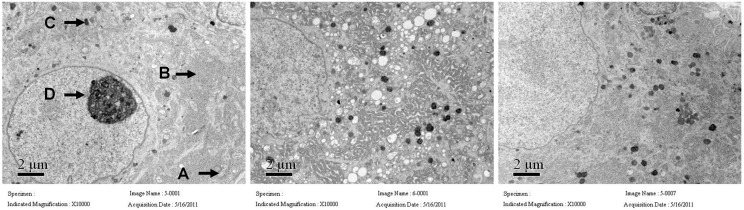
Transmission electron microscopy of major pelvic nerve ganglion in normal, DM placebo (untreated) and ICA II-treated group rat corpus cavernosum (A: mitochondria, B: mitochondria, C: mitochondria, D: mitochondria). The rough endoplasmic reticulum is highly dilated with degranulation, the mitochondria are swollen with vacuolization; the crista mitochondrion dissolved and fragmented, and the lysosome decreased in the DM placebo group. The microstructure was significantly improved in the ICA II-treated diabetic group compared to the DM placebo group.

We detected the microstructure by transmission electron microscopy of the normal control group, DM placebo group (untreated) and ICA II group. In the diabetic group the rough endoplasmic reticulum appeared highly dilated with degranulation and the mitochondria was swelled and displayed vacuolization; the crista mitochondrion was dissolved and fragmented, and also the lysosome was increased compared to the normal control group. The microstructure was significantly improved in the ICA II-treated diabetic groups compared to the DM placebo group (Figure 5).

**Figure 6:**
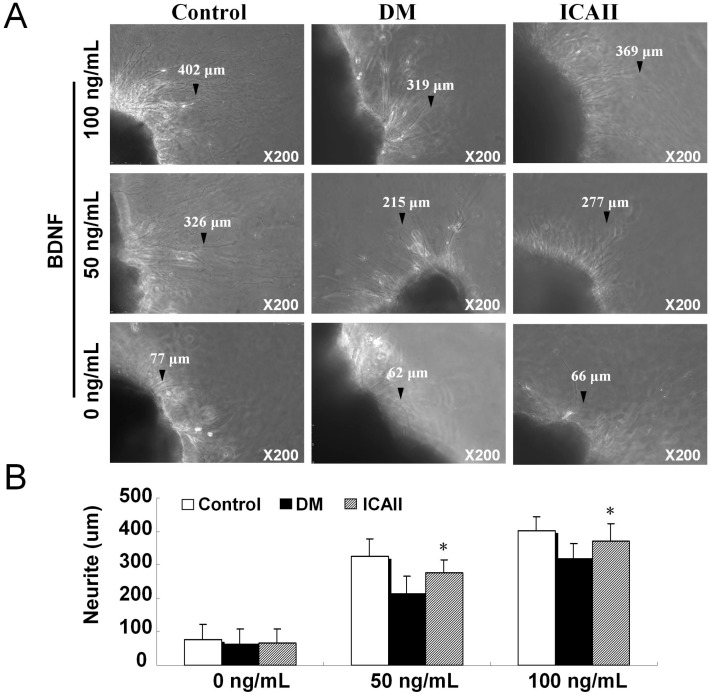
Neurite lengths of major pelvic ganglia treated with brain-derived neurotrophic factor (BDNF) cultured for 48 h in normal control and diabetic rat groups (the black arrows showed the typical neurite). (**A**) Representative photographs of major pelvic ganglia neurite with 0/50/100 ng/mL BDNF; (**B**) Average neurite length of major pelvic ganglia analysis results. Each bar depicts the mean values (±standard deviation) from *N* = 10 animals per group. * *p* < 0.01 in the ICA II-treated group compared with DM placebo group.

The neurite outgrowth length was measured in the cultured major pelvic nerve ganglion from the normal control group, DM placebo group (untreated) and ICA II-treated group. Paired comparisons were made between the major pelvic nerve ganglion derived from the normal control group, DM placebo group (untreated) and ICA II-treated group rats at the treatment of 0, 50, and 100 ng/mL BDNF at the 72 h time point, all major pelvic nerve ganglions treated with ICA II-treated had significantly longer average neurite length compared to DM placebo group (Figure 6).

## 3. Discussion

ED has a similar or greater predictive value for cardiovascular events than traditional risk factors including smoking, hyperlipidemia, and family history of myocardial infarction [20]. In the early course of diabetes, intracellular hyperglycemia causes abnormalities in blood flow and increased vascular permeability. DM-induced ED is a common diabetic complication and ED can be an early sign of diabetes in men who have not yet been diagnosed. Erectile dysfunction has a predictive value for cardiovascular events that is comparable to or better than that of traditional risk factors [21]. There are various causes for organic ED, most of which involve damage to nerves. We investigated the neuropathy changes in the corpus cavernosum of rats and the effects of ICA II on improving neuropathy.

Icariin has numerous potential applications in different fields of medicine However, it is best known for its putative role in potentiating sexual function by inhibition of PDE5 [22,23], We found that ICA II could alter corpus cavernosum fibrous-muscular pathological structure in DM rats which could be regulated by the TGFβ1/Smad2/CTGF and NO-cGMP signaling pathways [17]. Also Zhang *et al.* [24] reported that ICA-II increases the intracellular cGMP through the enhancement of nNOS expression and NOS activity in rat corpus cavernosum tissue *in vitro*. We found that the number of nNOS-positive nerve fibers in the dorsal penile nerves was significantly greater in the ICA II-treated group compared to the untreated diabetic animals.

As we reported before, fibrosis, accompanied with decreased smooth muscle, endothelial deterioration and neuropathy, was an important pathological process in diabetic ED models [17]. We found that penile tissues from diabetic rats showed decreased α-SMA, nNOS and eNOS expression in the cavernous sinusoids relative to the normal control group, and that ICA II ameliorated the endothelial cell and the smooth muscle cell. 

Currently there are no reports concerning the microstructure of the major pelvic ganglion, the density of the dorsal nerve bundle of penis or the ratio of the Schwann cell nucleus to the dorsal nerve bundle. In our study, we firstly reported that we performed the test of transmission electron microscopy of the major pelvic ganglion in normal control, DM placebo group (untreated) and ICA II-treated group. We found that the rough endoplasmic reticulum appeared highly dilated, and with degranulation, the mitochondria swollen and with vacuolization, crista mitochondrion dissolved and fragmented, and the lysosome decreased in the DM placebo group. However, the rough endoplasmic reticulum appeared significantly less dilation and degranulation in the ICA II-treated diabetic group. Also ICA II-treated diabetic rats had a higher density of dorsal nerve bundle compared to diabetic control group.

Cultured major pelvic nerve ganglion is an important and almost indispensable tool for the study of physiology and pathophysiology of neuropathy, Lin *et al.* [25,26] reported that BDNF and VEGF promote neurite growth from cultured MPG; BDNF promotes MPG neurite growth primarily by activating the JAK/STAT pathway. In our study all major pelvic nerve ganglion treated with ICA II had significantly longer average neurite length when compared to the DM placebo group; this effect was dose dependent, with greater concentrations leading to greater mean neurite length.

The efficacy of ICA II was not fully assessed in this study, thus, our data are limited in that functional assay. While this type of investigation is challenging, it should be a consideration for future work. To clarify the precise role of ICA II in diabetes related erectile impairment, further investigation on mechanisms (e.g., AGEs, inflammation *etc.*) of ICA II on diabetic ED are recommended.

## 4. Experimental Section

### 4.1. Preparation of ICA II

ICA II was bio-transformed from icariin by the action of β-glucosidase and the optimum reaction conditions as follows: 50 °C, 0.2 M disodium hydrogen phosphate and citric acid buffer system (pH 6.0); the ratio of icariin/enzyme was 1:1 and the reaction time was 5 h [27]. The purity of bio-transformed ICA II was 99.06% assayed by HPLC analysis.

### 4.2. Animals

A total of 36 male 8 week-old Sprague-Dawley rats weighing 200–250 g were randomly divided into three groups (normal control, diabetic and icariside II) in this study. The experiments performed were approved by the Institutional Animal Care and Use Subcommittee of our university. Rats were fasted for 16 h, and injected intraperitoneally with freshly prepared STZ (Sigma Chemical Co, St. Louis, MO, USA) (60 mg/kg) in diabetic and ICA-II groups, vehicle (0.1 mol/L citrate-phosphate buffer, pH 4.5) in normal control group. The ICA II was administrated at the dose of 5 mg/kg per day via gastric gavage (totally 2 mL) according to references for twelve weeks. Blood glucose levels were assayed 72 h after STZ or vehicle injection, at a regular interval of every week throughout the study, and immediately prior to euthanasia. Blood samples were obtained by tail prick, and blood glucose concentration measured using a blood glucose meter (B. Braun, Melsungen AG, Germany). Only those rats with fasting glucose concentrations ≥300 mg/dL were included in the diabetic group. 

Twelve weeks after induction of diabetes, we evaluated erectile function by cavernous nerve electrical stimulation, and the penis was harvested for histological examination (immunohistochemical stains) (each group contained 12 animals and for each animal at least 5 slides from different areas of the organ were examined). Separate cavernous specimens were used for Western blot. The major pelvic ganglions were harvested for culture and transmission electron microscopy.

### 4.3. Measurement of Erectile Function

The rats from each group were anesthetized with 5% sodium pentobarbital intraperitoneally. The major pelvic ganglion, cavernous nerves, and pelvic organs were exposed through a laparotomy incision. The skin overlying the penis was removed, and the penile crus was exposed by removing part of the overlying ischiocavernous muscle. Two 23-gauge needles connected to the PE-50 tube with heparinized saline (250 IU/mL) were carefully inserted into the crus and left carotid artery respectively. The other end of the PE-50 tube was connected to Biopac Systems MP150 (BIOPAC Systems Inc., Goleta, CA, USA). The cavernous nerve was exposed as described, and electrostimulation (12 Hz, pulse width 5 ms, 1/2.5/5 V, duration 60 s) of the cavernous nerve was applied with a stainless steel bipolar hook electrode (Concentric ndl electrd 30 mm TP). The mean arterial pressure (MAP, calculated according to the formula diastolic blood pressure + [(systolic blood pressure − diastolic blood pressure)/3] was measured concurrently. The ratio of maximal intracavernous pressure (ICP, mmHg) to mean arterial pressure (MAP, mmHg) was calculated to normalize for variations in systemic blood pressure [16,17].

### 4.4. Immunohistochemistry and Immunofluorescence

After ICP/MAP assessment, penile tissue was harvested and immersed in neutral buffered formalin containing 4% formaldehyde for a period of 6 h. Specimens were subsequently embedded in paraffin and sectioned at 5 μm using a rotor microtome. 

For immunohistochemistry and immunofluorescence (*N* = 10 per group), sections (5 µm) were deparaffinized and hydrated by sequential incubations in xylene and ethanol. After washing in 3× PBS for 5 min, the sections were blocked with 3% H_2_O_2_ for 10 min. The tissue sections were incubated with antibody to Neuronal Nitric Oxide Synthase (nNOS, Abcam, Cambridge, MA, USA; 1:500), Nerve Growth Factor, (NGF, Abcam, Cambridge, MA, USA; 1:800) Neurofilament (Abcam, Cambridge, MA, USA; 1:800) followed by the use of the Histostain^®^-Plus Kit (Zymed Laboratories, San Francisco, CA, USA). After the hybridization of secondary antibodies, and DAPI staining for the cell nucleus, the sections were observed at the fluorescence microscope (Leica DM 6000 Laser Station, Wetzlar, Germany). The analysis was performed to evaluate the intensity of Neuronal Nitric Oxide Synthase, Neurofilament staining by the use of Image-Pro plus software (version 6.0, Silver Spring, MD, USA) [16,17,25]. 

### 4.5. Western Blot

Cellular protein samples were prepared by homogenization of penile tissue in a lysis buffer containing 1% IGEPAL CA-630, 0.5% sodium deoxycholate, 0.1% sodium docecyl sulfate, aprotinin (10 μg/mL), leupeptin (10 μg/mL), and phosphate-buffered saline (PBS). Cell lysates containing 20 μg of protein were electrophoresed in sodium docecyl sulfate polyacrylamide gel electrophoresis and then transferred to a polyvinylidene fluoride membrane (Millipore Corp, Bedford, MA, USA). The membrane was blocked with 5% skimmed milk for 1 h at room temperature and incubated overnight at 4 °C with primary antibody (1:1000) against β-actin (Santa Cruz Biotechnology; San Francisco, CA, USA), (1:1000) nNOS (Abcam, Cambridge, MA, USA), and (1:1000) Nerve Growth Factor, (NGF, Abcam, Cambridge, MA, USA). After the hybridization of secondary antibodies, the resulting images were analyzed with ChemiImager 4000 (Alpha Innotech Corporation, San Leandro, CA, USA) to determine the integrated density value of each protein band. Results were semi-quantified by densitometry (*N* = 6 per group) [16,17].

### 4.6. Transmission Electron Microscopy

Specimens of major pelvic ganglion and dorsal nerve bundle (*n* = 8) were fixed with glutaraldehyde (3% in 0.1 mol·L^−1^ cacodylate buffer, pH 7.4) for 30 min at 4 °C, then post fixed in osmium tetroxide (O_S_O_4_) and embedded in Epon-812; 0.1-mm-thin sections were stained with uranyl acetate/lead citrate and viewed in a JEM1230 TEM (JEOL, Tokyo, Japan).

### 4.7. Major Pelvic Nerve Ganglion Tissue Culture in Vitro

The major pelvic ganglion tissue of normal control group, DM placebo group (untreated) and ICA II group (*n* = 12) were cultured *in vitro*, stimulation with different concentrations of brain derived neurophic factor (BDNF) and the length of neurite outgrowth was measured [25]. 

Each major pelvic ganglion tissue specimen was divided into two sections. After PBS rinsing, the freshly dissected major pelvic ganglion fragment was placed on a coverslip, to which a 14 µL drop of growth-factor-reduced Matrigel™ (Becton Dickinson, Mountain View, CA, USA) had been added and kept in liquid form using a cold 35-mm plastic culture dish on ice. The growth factor reduced Matrigel™ was polymerized (5 minute incubation at 37 °C) and 2 mL of serum-free RPMI-1640 added. Tissue fragments from the normal control group, DM placebo group (untreated) and ICA II group were treated at various concentrations of BDNF for 3 days (0, 10, 100 ng/mL). Ganglial cultures were maintained at 37 °C in a humidified atmosphere with 5% CO_2_. Photographs of neurite growth at 6 days were captured using a Nikon DXM 1200 digital still camera attached to Leica Laborluxmicroscope and ACT-1 software (Nikon Instruments Inc., Melville, NY, USA). Digital images were analyzed using Image-Pro Plus software to determine the longest neurite length per specimen. Mean maximal neurite length was calculated for the control, diabetic and ICA II group by averaging the longest neurite length from each individual specimen.

### 4.8. Statistical Analysis

Results are expressed as means ± standard deviation. One-way ANOVA followed by Bonferroni multiple comparisons’ test was used to evaluate whether differences between groups were significant. All calculations were performed using SPSS statistical software (version 13.0, SPSS, Chicago, IL, USA). Probability values of less than 5% were considered significant.

## 5. Conclusions

In summary, ICA II shows promise in the treatment of diabetes-related ED. This activity is likely modulated by preservation/recovery of nNOS and NGF activity. The corpus cavernosum pathological change of dorsal nerve bundle and the major pelvic nerve ganglion may play a critical role in ICA II-related recovery of erectile capacity in diabetic rats.
